# Outcomes associated with matching patients' treatment preferences to physicians' recommendations: study methodology

**DOI:** 10.1186/1472-6963-12-1

**Published:** 2012-01-03

**Authors:** Nasir Umar, David Litaker, Marthe-Lisa Schaarschmidt, Wiebke K Peitsch, Astrid Schmieder, Darcey D Terris

**Affiliations:** 1 Mannheim Institute of Public Health, Social and Preventive Medicine and the Competence Center for Social Medicine and Occupational Health Promotion, Medical Faculty Mannheim, University of Heidelberg, Ludolf-Krehl-Str. 7-11, 68167 Mannheim, Germany; 2 Departments of Medicine, Epidemiology and Biostatistics, Case Western Reserve University, Cleveland Ohio, USA; 3 Department of Dermatology, University Medical Centre Mannheim, University of Heidelberg, Theodor-Kutzer-Ufer 1-3, 68135 Mannheim, Germany; 4 Department of Health Policy and Management, College of Public Health, University of Georgia, Athens, Georgia, USA

**Keywords:** Patients' preferences, Patients' satisfaction, Psoriasis, Psoriasis treatment

## Abstract

**Background:**

Patients often express strong preferences for the forms of treatment available for their disease. Incorporating these preferences into the process of treatment decision-making might improve patients' adherence to treatment, contributing to better outcomes. We describe the methodology used in a study aiming to assess treatment outcomes when patients' preferences for treatment are closely matched to recommended treatments.

**Method:**

Participants included patients with moderate and severe psoriasis attending outpatient dermatology clinics at the University Medical Centre Mannheim, University of Heidelberg, Germany. A self-administered online survey used conjoint analysis to measure participants' preferences for psoriasis treatment options at the initial study visit. Physicians' treatment recommendations were abstracted from each participant's medical records. The Preference Matching Index (PMI), a measure of concordance between the participant's preferences for treatment and the physician's recommended treatment, was determined for each participant at t_1 _(initial study visit). A clinical outcome measure, the Psoriasis Area and Severity Index, and two participant-derived outcomes assessing treatment satisfaction and health related quality of life were employed at t_1_, t_2 _(twelve weeks post-t_1_) and t_3 _(twelve weeks post-t_2_). Change in outcomes was assessed using repeated measures analysis of variance. The association between participants' PMI scores at t_1 _and outcomes at t_2 _and t_3 _was evaluated using multivariate regressions analysis.

**Discussion:**

We describe methods for capturing concordance between patients' treatment preferences and recommended treatment and for assessing its association with specific treatment outcomes. The methods are intended to promote the incorporation of patients' preferences in treatment decision-making, enhance treatment satisfaction, and improve treatment effectiveness through greater adherence.

## Background

Patient-centered care has been defined as being "respectful of and responsive to individual patient preferences, needs and values and ensuring that patient values guide all clinical decisions" [1,2]. Patient-centered care fulfills the obligation of healthcare providers to place the interest of patients first and is associated with improved patient satisfaction, quality of life and better treatment adherence [1,3]. Although current debate exists around the definition of patient-centered care, matching preferences for care with the treatment provided is thought to be one of its key attributes [2,4].

Evidence supports the potential value of matching patients' preferences to treatment recommendations [4,5]. For example, positive treatment outcomes such as increased satisfaction with treatment and health-related quality of life have been demonstrated when patient preferences for treatment attributes were incorporated into treatment decision-making [5,6]. Matching patients' preferences for involvement in treatment decision-making to their actual level of involvement has also been associated with greater satisfaction with care processes and reduced levels of anxiety once treatment begins [5]. Insights from these studies are somewhat limited, however, by the methods used to elicit preferences. A simple binary approach (i.e. stating a preference for treatment A over treatment B), for example, fails to conform to traditional health economic practices in which measurement of the *strength *of patients' preferences for a specific treatment is thought to have greater meaning, and thus greater utility in decision-making. Moreover, the binary approach described fails to reflect the trade-offs made with respect to differing treatment attributes that may drive ultimate treatment choice. In addition, a large number of studies comparing patients' versus physician treatment preferences use hypothetical scenarios, which provide few insights into the real-world choices that patients actually encounter [7].

Patients' preferences for treatment have been shown to affect patients' treatment satisfaction [6,8]. Improvement in treatment satisfaction has been associated with patients' treatment adherence; patients' treatment adherence is considered necessary for achieving optimal treatment outcomes [8-11]. Thus, the conceptual model informing this work suggest that the incorporation of patients' treatment preferences in treatment decision making may influence patients' clinical (i.e. disease severity) and Health Related Quality of life (HRQoL) outcomes through greater satisfaction and adherence with physician treatment recommendations. Using a prospective cohort study design, our methods aimed to test the hypothesis that a closer match between physicians' treatment recommendations and patients' treatment preferences may lead to reduction in disease severity, less impairment in HRQoL, and improvement in treatment satisfaction.

Tools that assess the strength of patients' preferences for treatment recommendations and potential trade-offs among treatment attributes are needed. The availability of such tools may enable more accurate assessment of the effects of preference matching on treatment outcomes. In this report, we describe the methods we used in a study that assessed the association of preference matching with objective clinical and participant-derived outcomes.

## Methods

### Setting

This study is being carried out in a bi-weekly outpatient psoriasis clinic in the Department of Dermatology, University Medical Centre Mannheim, University of Heidelberg, a regional 'Competence Centre for Psoriasis.' The psoriasis clinic is a primary, secondary and tertiary care referral centre, to which patients are referred by family physician, general medical practitioners, general internist, dermatologists and other community hospitals from the Metropolitan Region Rheine-Necker. The clinic therefore attends to population of patients with wide spectrum of the disease. Approximately 250 to 300 patients with moderate to severe psoriasis attend these clinics annually, making it a site at which it would be feasible to recruit participants with a range of clinical characteristics and disease severity.

### Participants

Study participants were patients attending the outpatient psoriasis clinic in the Department of Dermatology, University Medical Centre Mannheim. University of Heidelberg. *Inclusion criteria: *participants were included if they were 18 years of age or older and were new or established patients in the Department of Dermatology. Each had physician-diagnosed moderate or severe psoriasis according to the criteria of the Committee for Medicinal Products for Human Use [12], i.e., a Psoriasis Area and Severity Index (PASI) ≥ 10, involvement of the head, the palmar or plantar surfaces, or psoriatic arthritis with any skin involvement and patients on systemic anti-psoriatic therapy. These criteria were purposefully selected to ensure that participants in the sample would require one or more treatments from a broad range of available options. *Exclusion criteria: *Participants were excluded if they were not able to complete the online survey independently or if they were unable to read and understand German (the language in which the survey and subsequent interviews were conducted).

### Recruitment

To recruit a consecutive sample, each patient with moderate or severe psoriasis attending dermatology outpatient clinics was approached. Two members of the research team were responsible for all aspects of recruitment, including the distribution of informational leaflets about the study, identification of potentially eligible participants, assessment of eligibility (i.e., applying inclusion/exclusion criteria) and obtaining informed consent. Once identified, eligible participants were approached before their appointment with the physician and invited to participate in the study. Informed consent was then obtained, a unique four-digit study identification number was assigned and the two study follow-up visits were scheduled (t_2 _(twelve weeks post-t_1_) and t_3 _(twelve weeks post-t_2_)). After completing an online survey (described below), participants proceeded to their medical appointment. Steps in recruitment and data collection are summarized in Table 1.

**Table 1:** Steps involved in participants' recruitment and data collection

Recruitment/ Data collection steps	Activities
1. Participant recruitment	Participants were approached at the outpatient clinic while waiting for their doctor's appointment. The setting was considered convenient for recruitment as it provided access to relatively large numbers of potential participants who fit the study's inclusion criteria. Further, since patients have to wait for their doctor's appointment at the outpatient clinic, asking participants to answer the survey during this waiting period was not viewed as imposing an undue burden on their time.

2. Informed consent	Patients who agreed to participate completed and returned a signed informed consent form.

3. Allocation of study identification number/initial screening	Participants who returned the signed consent forms and were considered eligible were assigned study identification numbers for anonymity. Appointment dates were also set for subsequent follow-up visits.

4. Administration of the survey	Participants' were guided to the room and computer where they completed the survey.

5. Doctor's appointment	After completing the survey, participants were directed to their respective doctor's appointment.

6. Data abstraction and forwarding	Participants' PASI scores and doctor's treatment recommendation were abstracted from the medical records and faxed to the study coordination center at the Mannheim Institute of Public Health (MIPH) for entry into a database.

7. Participant screening	Inclusion and exclusion criteria were further applied at this stage, using the abstracted data, to screen participants for eligibility.

8. Non-eligible patients	Participants considered non-eligible were not followed for subsequent study visits and their records were deleted.

9. Eligible patients	Eligible patients were followed for subsequent study visits and their records were stored according to data protection laws.

10. **t_1 _**(initial study visit)	Initial study visit data was collected.

11. **t_2 _**(12 weeks after **t_1_**)	First follow-up visit data was collected.

12. **t_3 _**(12 weeks after **t_2_**)	Second follow-up visit data was collected.

13. Study coordination and data storage at MIPH	The survey data forwarded from the study site (dermatology department) was securely stored at the MIPH.

### Data elements

The **primary independent **variable was the Preferences Matching Index (**PMI**), a measure of concordance developed in three steps: **Step 1 **involved the elicitation of participants' preferences for available treatment options in terms of their "attributes" (i.e., processes or potential outcomes of treatment [see Table 2][13]) and attribute "levels" or "categories" (i.e., the possible forms a treatment might take). In **Step 2**, physicians' treatment recommendations were abstracted from participants' medical records. **Step 3 **entailed the calculation of the PMI using a process of conjoint analysis to quantify concordance between participants' treatment preferences and the treatment recommended at the initial study visit at (t_1_) by the physician. The PMI was determined only at t_1 _(initial study visit). Each step is described below in detail.

**Table 2:** Profile of treatment attributes and attribute levels

Treatment Attribute	Attribute Levels (categories)
**Treatment duration^a^**	**Each treatment will take:** • 5 minutes to complete. • 15 to 30 minutes to complete. • 1 hour to complete. • 2 hours to complete.

**Treatment frequency^a^**	**My treatment will occur:** • Once every three months. • Once every two weeks. • Two times each week. • Twice daily.

**Treatment cost^a^**	**I will have to pay:** • Nothing to cover the cost of my treatments. • An additional 50 € per month to cover the cost of my treatments. • An additional 100 € per month to cover the cost of my treatments. • An additional 200 € per month to cover the cost of my treatments.

**Treatment location^a^**	**My treatment will take place:** • At home. • At home with follow-up at my local doctor's office. • At an outpatient clinic. • While I stay in the hospital for three weeks.

**Treatment delivery method^a^**	**My treatment will occur by:** • Applying medication on my skin. • Taking Tablets. • Having an injection/intravenous infusion. • Light therapy.

**Magnitude of beneficial effect^b^**	**I will likely experience:** • Almost a 100% reduction in my psoriasis plaques. • About a 75% reduction in my psoriasis plaques. • About a 50% reduction in my psoriasis plaques. • About a 25% reduction in my psoriasis plaques.

**Duration of beneficial effect^b^**	**The improvement in my psoriasis will last for:** • 1 year or more after completing all of my treatments. • 6 to 8 months after completing all of my treatments. • 3 to 5 months after completing all of my treatments. • 2 weeks after completing all of my treatments.

**Probability of side effects^b^**	**There is:** • Almost a 100% chance that I will experience side effects from the treatment. • About a 50% chance that I will experience side effects from the treatment. • About a 10% chance that I will experience side effects from the treatment. • Less than 1% chance that I will experience side effects from the treatment.

**Probability of beneficial effect^b^**	**I have:** • Almost a 100% chance of experiencing a significant reduction in my psoriasis. • About an 80% chance of experiencing a significant reduction in my psoriasis. • About a 60% chance of experiencing a significant reduction in my psoriasis. • About a 40% chance of experiencing a significant reduction in my psoriasis.

**Reversibility of side effects^b^**	**If side effects occur, there is:** • Almost 100% chance that I will completely recover once my treatments are stopped. • About an 80% chance that I will completely recover once my treatments are stopped. • About a 60% chance that I will completely recover once my treatments are stopped. • About a 40% chance that I will completely recover once my treatments are stopped.

**Side effect severity^b^**	**I may experience:** • Temporary, minor discomfort on my skin. • Constant, moderate discomfort on my skin. • Temporary, moderate side effects that can effect more than my skin. • Severe side effects that can effect more than my skin.

Note: (**^a^**) = Process attributes and levels; (**^b^**) = Outcome attributes and levels

#### Elicitation of patients' preferences (Step 1)

'Patients' preferences' here refers to the value patients attach to different treatment attributes when faced with treatment options. A range of potentially appropriate and currently available psoriasis treatments modalities were identified by using the 'German evidence-based guidelines for the treatment of psoriasis' [14] and by consultations with clinical experts (AS and WP). Process and outcomes of currently available treatments were decomposed into attributes and attribute levels or categories. This process was guided by review of studies that assessed preferences for psoriasis treatments [5,6,15].

Four attribute levels or categories were specified for each treatment attribute (Table 2)[13]. Although a large number of attribute levels could have been developed, we decided to limit confine the number to four categories to limit respondent burden and avoid information overload. We further refined attribute categories for clarity of content using comments from the participants, following a pilot of the conjoint analysis exercise described below. Treatment attributes and attribute categories were labeled to distinguish elements of the process of treatment from those resulting from the care (outcomes). Examples of process attributes included the 'delivery method' (mode of drug administration) or 'location of treatment'. An example of attribute *categories *for the process-related treatment attribute 'location of treatment' included: 'treatment at home', 'treatment at the local doctor's office', 'treatment at an outpatient clinic', and 'treatment at an inpatient clinic'. Examples of outcome attributes included the 'severity of potential side effects', 'possibility of beneficial effect' and 'reversibility of side effects' that may result from a particular psoriasis treatment modality. A full list of attributes and attribute categories is provided in Table 2[13].

We used 'choice-based conjoint analysis' (CBC) to measure participants' preferences for specific psoriasis treatments. This method best simulates the way people make everyday choices when faced with multiple options [16] and has the additional advantages of being previously validated, easy to use, and efficient in assessing patients' preferences for health care and their treatment priorities [16]. To conduct our analysis, we used survey design software (Sawtooth, Inc., Sequim, WA) to present participants with twelve pair-wise comparisons comprised of random combinations (see Table 3)[13] of the profiled attribute categories (twelve random combinations of attribute categories per pair). In a final analytical step, "preference scores" (partworth utilities values) were generated for every participant for each attribute level or category of psoriasis treatment option, with higher scores indicating a greater preference. It is important to note that even though a subset of potential treatments were randomly selected and presented to the participant, the software algorithm (using an orthogonal design) is programmed to extrapolate preference values for all possible treatments and treatment levels for each study participant.

**Table 3:** Example of treatment scenarios presented to the study participants in the conjoint analysis survey

**Imagine that you will be actively treating your psoriasis for the next three months. From each pair of treatment options A and B, please pick which treatment you will like to participate in**.	
**Option A** My **treatments **will take place **at home**. My **treatments **will occur **twice daily**. Each **treatment **will take **one hour **to complete.	**Option B** My **treatments **will take place while I **stay **in the **hospital **for **three weeks**. My **treatment **will occur **once every three months**. Each **treatment **will take **15 to 30 **minutes to complete.
I may **experience **constant, moderate **side effects **that can affect **more than my skin**.	I may **experience **constant, **minor discomfort on** **my skin**.
I have about an **80% chance **of experiencing a significant **reduction **in my **psoriasis plaques**.	I have almost a **100% chance **of experiencing a significant **reduction **in my **psoriasis plaques**.
The **improvement **in my psoriasis will last **for 3 to 5 months after **completing all of my **treatments**.	The **improvement **in my psoriasis will last **for 3 to 5 months after **completing all of my **treatments**.

#### Abstraction of physician-recommended treatments (Step 2)

A member of the research team (MS) trained in the interpretation of data documented in medical records was responsible for abstracting data on the actual treatment modalities recommended by physicians for each participant. Participants' medical records were retrieved after their clinical visit at t_1 _and data were abstracted using a standardized data collection form and entered into a database. Accuracy of data entry was confirmed by review at each subsequent study visit of all previously recorded data. In the event of multiple treatment recommendations at t_1_, all were recorded.

#### Determining the level of concordance (Step 3)

First we identify participants'-preferred treatments and most preferred attribute categories with the highest scores; we also identified participants' least preferred treatment and least preferred attribute categories with the lowest scores from the conjoint analysis described above. The same process was followed for identifying the scores associated with the physician-recommended treatments, and their associated attributes, for each participant.

To illustrate, if Table 4 represents preference scores from a conjoint analysis for a hypothetical participant presented with three randomly selected treatment attributes, the participant appears to most prefer light therapy treatment (preference score = 44) at an outpatient clinic (preference score = 19) lasting between 15-30 minutes (preference score = 27). If the treatment recommended by the physician for this participant was 'methotrexate tablets', the attribute categories ascribed to this treatment would be: "tablets", "taken at home" and "5 minutes" for anticipated treatment type, location, and anticipated duration, respectively. These attribute categories correspond to participant-derived preference scores of 10, 17 and 23 (Table 4).

**Table 4:** Example of preferences scores (partworth utilities) for treatment attributes and attribute levels for a hypothetical respondent

Treatment attributes	Attribute categories	Preference score (utilities)
Attribute 1. Treatment delivery method	Category 1. Topical	-74
	Category 2. Tablets	10
	Category 3. Injection/infusion	20
	Category 4. Light therapy	44

Attribute 2. Treatment location	Category 1. At home	17
	Category 2. At local doctor's office	2
	Category 3. At outpatient clinic	19
	Category 4. Hospital stay	-34

Attribute 3. Treatment duration	Category 1. 5 minutes to complete	23
	Category 2. 15-30 minutes to complete	27
	Category 3. 1 hour to complete	-26
	Category 4. 2 hours to complete	-24

Next, scores for treatment attribute categories most preferred by the participants (attribute categories with the highest preferences score) and attribute categories least preferred by the participants (attribute categories with lowest preference scores) were summed. Further, scores for physician recommended treatment were summed. For example, the sum of preference scores for our hypothetical participants' most preferred treatment and least preferred treatment in the example above is 90 (= 44+19+27) and -134 (= -74-34-26) respectively, while the sum of preference scores for the physician-recommended psoriasis treatment is 50 (= 10+17+23).

**Figure 1:**
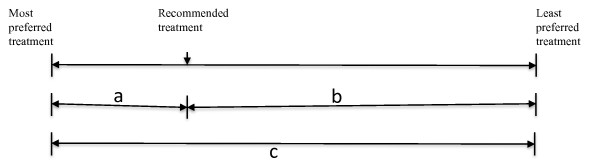
**Illustration of the scaling used to determine the Preference Matching Index**.

We then constructed a scale with two end points: the participants' most preferred treatment and the participants' least preferred treatment (Figure 1). To determine the PMI, the range between the preference scores for recommended treatment and the least preferred treatment was divided by the range between the most preferred treatments and the least preferred treatment or b/c (Figure 1). In the example, the PMI is therefore [50-(-134)/90-(-134)], representing the concordance between the preference scores for the physician treatment recommendations and preference scores for the participants' preferred treatment. The PMI ranges between 0 (no preference concordance) and 1 (complete preference concordance). To ensure data quality and the accuracy of the PMI computed, we repeated calculations at each stage. In the rare instances in which discrepancies were found, we returned to the original data to confirm and re-enter values.

The **dependent variables **in our analysis consisted of both objective clinical and participant-derived outcome measures (Table 5). The dependent variables were assessed t_1_, t_2 _(twelve weeks post-t_1_) and t_3 _(twelve weeks post-t_2_). Twelve weeks are known to be sufficient to judge the short-term effectiveness of a psoriasis treatment, but we specifically wanted to look at longer-term outcomes at 24 weeks which may better represent sustainable treatment adherence [17]. Our analyses therefore focused on changes observed between t_1 _and t_3_, although additional analyses (not described here) were also performed for changes observed between t_1 _and t_2_.

**Table 5:** Variables, measurement instruments and data sources

Variable	Measurement Instrument	Data Source	Data Collection Period
**Independent Variable**			

PMI	Conjoint analysis survey/Doctors' recommendations	Participants	**t_1_**

PMI	Doctors' recommendations	Participants' medical records	**t_1_, t_2_, t_3_**

**Dependent Variable**			

PASI scores	Physician-assessed PASI [17]	Participants' medical records	**t_1_, t_2_, t_3_**

TSQM scores	Self-reported TSQM questionnaire [18]	Participants	**t_1_, t_2_, t_3_**

DLQI scores	Self-reported DLQI questionnaire [19]	Participants	**t_1_, t_2_, t_3_**

**Confounders**			

Demographic factors	Standard German demographic questionnaire	Participants	**t_1_**
Sex Age Partnership Living with a partner/living alone Education Highest educational attainment Income Net monthly household income Number of household members Employment status Full-time Part-time Not working			

Treatment factors	Self-reported questionnaire	Participants	**t_1_**
Currently receiving psoriasis treatment or not Type of psoriasis treatment (topical, UV therapy, Tablets, injections/infusions)			

Disease-related factors	Self-reported questionnaire	Participants	**t_1_**
Time since diagnosis Co-morbidities Psoriatic arthritis Depression Allergy High blood pressure Cardiovascular disease Hyperlipidemia Chronic lung disease, asthma Liver disease Diabetes Cancer			

Note: DLQI = Dermatology Quality of Life Index; PASI = Psoriasis Area and Severity Index; TSQM = Treatment Satisfaction Questionnaire for Medication; t_1 _= initial study visit; t_2 _= first follow-up study visit twelve weeks after t_1_; t_3 _= second follow-up study visit twelve weeks after t_2_

The **primary dependent variable **was the change in PASI score from t_1 _to t_3_. The PASI is a psychometrically valid and reliable instrument routinely applied in dermatology to assess psoriasis severity and gauge treatment effects [17]. PASI combines assessment of the severity of psoriasis lesions and the area affected into a score ranging from 0 (no disease) to 72 (maximal disease), with scores ≥ 10 reflecting moderate to severe disease [17].

The **secondary dependent variables **included change in patients' satisfaction with treatment from t_1 _to t_3 _measured by the Treatment Satisfaction Questionnaire for Medication (TSQM) and change in self-reported health-related quality of life (HRQL) from t_1 _to t_3 _measured by the Dermatology Quality of Life Index (DLQI). TSQM is a 14-item, psychometrically validated instrument [18]. The DLQI, a validated questionnaire, is one of the most widely used instruments to assess the health-related quality of life of patients with skin conditions. DLQI scores range from 0 to 30, with higher scores indicating greater impairment in skin disease-specific quality of life [19].

We identified **potential confounding characteristics **including sex (male, female); age (measured in years); partnership status (i.e., living with a partner, not living with a partner, widowed); employment status (i.e., full-time, part-time, not working), highest educational attainment (i.e., no school, primary school equivalent, secondary school equivalent, post-secondary school training, university, post-university) and net annual household income (measured in Euros) [20,21]. Based on previous work, we measured a number of factors that might moderate participants' satisfaction and compliance with treatment, including treatment history (i.e., previously prescribed treatments) and disease-related factors (i.e., time since diagnosis and co-morbidities [e.g. depression: yes/no]) [21].

### Data sources/data collection

As previously mentioned, data were obtained from two sources: participants' survey responses and physicians' notations in their medical records (Table 5). The survey, developed using 'Sawtooth Survey Software for Online Interviewing' (Sawtooth, Inc., Sequim, WA), was administered in the dermatology clinic using either a desktop computer or laptop located in a separate room away from the waiting area. A member of the research team was available to demonstrate how the survey instrument functioned. Data collected via the online survey was stored in a secure server and accessed for analysis via the Internet.

### Analytic strategy

#### Design

We utilized a prospective cohort study design to assess the association of the concordance between patients' preferences to physician-recommended treatments with subsequent treatment outcomes.

#### Sample size

To detect a moderate-sized change in our primary outcome measure between t_1 _and t_3_, we set an effect size (f^2^) of 0.15 [12], α = 0.05 and β = 0.20. Using these parameters, we estimated that a minimum sample size of 200 was needed. Adjusting for a possible dropout rate of 20% at both the t_2 _and t_3 _follow-up visits, we increased the required sample size to 240 patients. We considered this recruitment goal to be attainable given the annual patient volume at the study site.

#### Specific aims, tasks and hypotheses

Our methods aimed to address the following:

• To develop the Preference Matching Index (PMI) as a novel metric that assesses the concordance between a physician's treatment recommendation and a participants' most preferred treatment.

• To evaluate the association between PMI scores and change in PASI over time.

***Hypothesis***: There will be a statistically significant negative association between participants' PMI and absolute change in PASI between t_1 _and t_3_.

• To evaluate the association between participants' PMI scores and non-clinical outcomes including change in satisfaction with treatment and Health Related Quality of Life (HRQL).

***Hypotheses***: There will be a statistically significant positive association between PMI and absolute change in measures of satisfaction with treatment between t_1 _and t_3_.

***Hypotheses***: There will be statistically significant negative associations between participants' PMI and absolute change in change in HRQL between t_1 _and t_3_.

#### Statistical analysis

Baseline data will be used to identify factors associated with participants' preferences and treatment satisfaction, HRQoL and disease severity in the study sample. Data of participants' lost to follow-up will examine the characteristics associated with dropout. Change in outcome measures across the three data collection points will be assessed using repeated measure analysis of variance. The relationship between participants' PMI scores and each of the three study outcomes will be evaluated using separate multivariate linear regressions models: the association of PMI with the objective clinical outcome (model 1) and the association of PMI with patient reported treatment satisfaction (model 2) and HRQL (model 3), controlling for known confounders (e.g. age, education, marital status and income) [22]. In sensitivity analysis, we will stratify our analysis by gender, and by new and established (old) patients; treatment experience (new or old) may affect patients' treatment preferences)[23].

#### Ethical and human subjects' confidentiality

The ethics committee of the Medical Faculty Mannheim, University of Heidelberg granted approval for the study (ID 2009-329E-MA). The methodology used in this study followed the principles of the Helsinki Declaration. Statistical analyses were performed using either Sawtooth software (Sawtooth, Inc., Sequim, WA) or SPSS statistical package version 19 (Chicago, IL).

## Discussion

In this paper, we describe the methods used to develop a measure of the concordance between patients' preferences for treatment and actual treatment recommendations. We also detail the methods used to assess the association of this measure with both objective clinical and patient-derived outcomes.

Substantial effort has been devoted to creating a health care environment in which the needs and preferences of patients are both acknowledged and incorporated into decision-making. Unfortunately, the tools available to support this effort are limited in both quantity and quality. Ideally these tools should be capable of assessing a broad spectrum of available medical interventions (i.e., the processes of care) and realistic outcomes of care. Conjoint analysis best simulates the way people make everyday choices when faced with multiple options and can help identify treatment features that drive patients' treatment preferences (9). Further, conjoint analysis has the additional advantages of being validated, easy to use, and increasingly recognized as an efficient way to assess patients' preferences for health care and their treatment priorities [16]. In addition, in routine clinical practice or research, the preference scores derived from the conjoint exercises can be compared with treatment recommendations from physicians to determine the extent of preference matching and sharing [16].

The methods we describe may be applied to decision-making for the management of other chronic diseases. Although such applications requires an initial investment of time and expertise in revision of the conjoint analysis survey, the introduction of new treatments (attributes and attribute levels) can be subsequently added with relative ease to data previously stored by the program. Our work uses psoriasis as an example of a common chronic disease, yet we fully expect that this methodology may be useful in promoting shared decision-making in the management of other diseases.

Mismatch between physicians' and patients' treatment preferences have been reported [24]. Mismatch between physicians' and patients' treatment preferences may results in patients receiving treatment they are dissatisfied with, which may affect their adherence to the recommended treatments [24]. Understanding the association of preference matching (captured by the PMI) with treatment outcomes may provide important insight on the potential value of using psoriasis patients' preferences in shared decision-making.

Based on the preference scores elicited from the conjoint analysis, it is possible for a patient to have equal or near equal preference for two different treatment options [25], if the two treatment options share certain attributes the patient value equally (e.g. a tablet and a cream can both be taken at home and their administration may take less time). This has important implication in treatment decision; it gives room for a more meaningful shared decision-making to happen. Physicians can acknowledge patients preferences, and still have the freedom to trade between different treatment attributes and attribute levels when making treatment recommendations.

Despite the strength of the methods we have described, a number of limitations should be acknowledged. Participants may, for example, report biased preferences when completing the conjoint exercise due to a 'dominant preference' for a particular attribute. That is, they may refuse to trade between treatment attribute categories because of strong feelings about the perceived nature of some treatments. Second, we have not measured adherence directly. The conceptual model informing this work suggests, however, that satisfied patients are more likely to adhere to recommended treatments and that this explains, to some extent, the presence of more desirable outcomes. Finally, some have suggested that the process of preference elicitation may influence subsequent reporting of treatment satisfaction or disease-specific quality of life. To guard against this, we assessed preferences before consultation and did not share these results with patients at any point during the study.

## Conclusion

We describe a novel method for measuring concordance between patients' most preferred treatments and physicians' recommended treatments and for assessing the association of concordance with treatment outcomes. Tailoring treatment recommendations to patients' preferences is an essential part of patient-centered care. Efforts to implement preference-based tailoring in routine clinical care may improve the quality of the interaction between patients and physicians, patients' adherence with treatment, satisfaction with treatment and clinical outcomes achieved. The methods reported here, although originally tested within the context of psoriasis treatment, have the potential to advance greater patient-centeredness in the management of other chronic diseases.

## Abbreviations

PMI: Preference Matching Index; HRQoL: Health Related Quality of Life; PASI: Psoriasis Area and Severity Index; TSQM: Treatment Satisfaction Questionnaire for Medication; DLQI: Dermatology Quality of Life Index.

## Competing interests

The authors declare that they have no competing interests.

## Authors' contributions

Study concept and design: NU, WKP, AS & DDT. Drafting of the manuscript: NU & DDT. Critical revision of the manuscript for important intellectual content: NU, DL, MS, WKP, AS & DDT. Administrative, technical, or material support: NU, MS, WKP, AS & DDT. Study supervision: NU, WKP & DDT. All authors read and approved the final manuscript.

## Pre-publication history

The pre-publication history for this paper can be accessed here:

http://www.biomedcentral.com/1472-6963/12/1/prepub
